# Cancers du pancréas au Centre National Hospitalier Universitaire de Cotonou: aspects épidémiologiques, diagnostiques, thérapeutiques et pronostiques

**DOI:** 10.11604/pamj.2021.39.18.26261

**Published:** 2021-05-06

**Authors:** Aboudou Raïmi Kpossou, Dansou Gaspard Gbessi, Freddy Houéhanou Rodrigue Gnangnon, Meissarath Modoukpè Ba Boukari, Rodolph Koffi Vignon, Comlan N´déhougbéa Martin Sokpon, Jean Sehonou

**Affiliations:** 1 Clinique Universitaire d´Hépato-gastroentérologie, Centre National Hospitalier Universitaire-Hubert Koutoukou Maga (CNHU-HKM), Cotonou, Bénin,; 2 Clinique Universitaire de Chirurgie Viscérale, Centre National Hospitalier Universitaire-Hubert Koutoukou Maga (CNHU-HKM), Cotonou, Bénin

**Keywords:** Cancer du pancréas, épidémiologie, traitement, pronostic, Bénin, Cancer of the pancreas, epidemiology, treatment, prognosis, Benin

## Abstract

**Introduction:**

le cancer demeure une cause majeure de décès dans le monde. Ce travail avait pour but d´étudier les caractéristiques épidémiologiques, cliniques, thérapeutiques et pronostiques des cancers du pancréas (CP) au Centre National Hospitalier Universitaire de Cotonou.

**Méthodes:**

il s´agissait d´une étude transversale descriptive et analytique, avec un recueil de données à la fois prospectif et rétrospectif sur une période de dix ans, allant du 1^e^ ^r^Octobre 2009 au 31 Octobre 2019.

**Résultats:**

sur 15.102 hospitalisations, 72 cas de CP avaient été recensés soit une fréquence hospitalière de 0,5%. L´âge moyen des patients était de 59 ans. La sex-ratio (H/F) était de 1,5. Le principal motif de consultation était la douleur abdominale. Plus de la moitié (51,4%) des patients avaient une maladie métastatique au moment du diagnostic. La preuve histologique d´adénocarcinome n´était disponible que dans 15,1% des cas. Le taux d´opérabilité était de 37,5% et le taux de résécabilité de 2,7%. La chimiothérapie essentiellement palliative était administrée à 13,9% de nos patients. Le coût moyen du traitement était de 955.882,4 FCFA (23,9 fois le Salaire Minimum Interprofessionnel Garanti au Bénin). La médiane de survie globale était de 6 mois. La létalité était de 86,9% (53/61), la survie à un an de 31,4%, et à 5 ans, nulle. La chirurgie palliative (p = 0,021) et la chimiothérapie (p = 0,023) amélioraient la survie des patients.

**Conclusion:**

le cancer du pancréas, en raison de ses signes peu spécifiques et de son évolution insidieuse, est souvent de diagnostic tardif. Le stade métastatique et les faibles possibilités thérapeutiques individuelles et institutionnelles contribuent à rendre le pronostic plus péjoratif.

## Abstract (en)

**Introduction:**

cancer is a major cause of death in the world. The purpose of this study is to evaluate the epidemiological, clinical, therapeutic and prognostic features of cancers of the pancreas (CP) at the National Hospital and University Center of Cotonou.

**Methods:**

we conducted a cross-sectional descriptive and analytical study with a prospective and retrospective data collection over a period of ten years, from 1 October 2009 to 31 October 2019.

**Results:**

out of 15.102 hospitalizations, we identified 72 cases of CP, reflecting a hospitalization rate of 0.5%. The average age of patients was 59 years. The sex-ratio (H/F) was 1.5. The main reason for consultation was abdominal pain. More than half (51.4%) of patients had metastatic tumor at the time of diagnosis. Histological evidence of adenocarcinoma was only reported in 15.1% of cases. The rate of operable patients was 37.5% while the rate of resectable patients was 2.7%. Palliative chemotherapy was given to 13.9% of patients. The average cost of treatment was 955.882,4 FCFA (23.9 times the Guaranteed Interprofessional Minimum Wage in Benin). Median overall survival was 6 months. Mortality rate was 86.9% (53/61), survival rate at one year was 31.4%, and zero at five years. Palliative surgery (p = 0.021) and chemotherapy (p = 0.023) improved patient survival.

**Conclusion:**

cancer of the pancreas, due to its non-specific signs and insidious outcome, is often diagnosed at a late stage. A metastatic tumor and the limited individual and institutional therapeutic possibilities lead to more pejorative prognosis.

## Introduction

Le cancer est une cause majeure de décès dans le monde. En 2018, le fardeau mondial du cancer s'élevait à 18,1 millions de cas dont 9,6 millions de décès [1]. Cette mortalité peut être imputable à certains cancers de mauvais pronostic tels que le cancer du pancréas (CP). En effet, le CP est l´un des cancers les plus létaux avec un ratio mortalité/incidence de 94% [1]. Il représente la 7^e^ cause de décès par cancer dans le monde [1] et reste le cancer digestif dont le pronostic est le plus défavorable, avec un taux de survie globale à 5 ans de 7 à 8% [2]. Ces chiffres alarmants s´expliquent en partie par l´absence de facteurs étiologiques spécifiques rendant difficile la prévention. De plus, le diagnostic est souvent tardif en raison de signes cliniques peu révélateurs voire absents au début de la maladie. A ces raisons s´ajoutent l´absence de marqueur biologique précoce et fiable disponible en pratique clinique quotidienne et le potentiel métastatique précoce, ainsi que l´agressivité de la tumeur [3]. Par ailleurs, à l´heure actuelle, le seul espoir de guérison d´un CP réside dans la chirurgie; cependant, celle-ci ne peut être instituée à visée curative que dans 15% des cas [4].

En Afrique, le CP représente la 14^e^ cause de décès par cancer avec 15,458 décès pour les 16,059 cas diagnostiqués en 2018 [1]. Au Bénin, la situation apparaît tout aussi préoccupante d´après les données 2014-2016 du registre des cancers de Cotonou qui classait le CP en 12^e^ position en termes de fréquence parmi les cancers dans les deux sexes [5]. Toutefois, peu d´études prospectives ont été réalisées sur le sujet dans la sous-région et au Bénin, malgré l´incidence de plus en plus croissante de cette pathologie et son pronostic péjoratif. Ces constats nous ont motivé à réaliser cette étude. Le but de ce travail était d´étudier les caractéristiques épidémiologiques, cliniques, thérapeutiques et pronostiques des cancers du pancréas au CNHU-HKM de Cotonou.

## Méthodes

Il s´agissait d´une étude transversale descriptive et analytique, avec un recueil de données à la fois prospectif et rétrospectif sur une période de dix (10) ans allant du 1^er^ Octobre 2009 au 31 Octobre 2019. Elle avait porté sur les patients atteints de CP dans les cliniques universitaires d´Hépato-gastroentérologie, de Chirurgie Viscérale et de Médecine Interne du CNHU-HKM. Le diagnostic de cancer du pancréas a été posé sur la base d´arguments cliniques, morphologiques et/ou histologiques. La survie globale a été déterminée comme étant la durée de la période à compter de la date du diagnostic jusqu´à la date du décès (de toute cause).

Nos données ont été colligées sur des fiches d´enquête et analysées à l´aide des logiciels Epi Data 3.1. et STATA 15. La méthode de Kaplan Meier était utilisée pour l´analyse de la survie. Le seuil de significativité était de 5%. Comme il s'agissait d'une étude en bonne partie rétrospective, il y avait des données manquantes. Ces patients étaient exclus des analyses pour les variables dont les données manquaient.

Sur le plan éthique, l´étude n´a pas été soumise à un comité local d´éthique. Néanmoins, le consentement éclairé verbal des participants a été obtenu pour la phase prospective de l´étude. Par ailleurs, la confidentialité et le respect de la personne humaine ont été observés lors du recueil et du traitement des données.

## Résultats

### Caractéristiques de la population d´étude

Nous avons recensé 72 cas de cancer du pancréas sur les 15102 admissions enregistrées dans les services ciblés au cours de la période d´étude soit une fréquence hospitalière de 0,5%. La fréquence moyenne annuelle est de 7,2 cas (72 cas sur 10 ans). L´âge moyen des sujets était de 59 ans avec un écart-type de 10,1 ans et des extrêmes de 30 ans et 85 ans. La fréquence maximale de CP était observée dans la tranche entre 60 et 69 ans (38,9%; n = 28). On notait une prédominance masculine (61,1%), avec une sex-ratio (H/F) de 1,5. Les ménagères et revendeurs étaient la classe professionnelle la plus représentée avec 37,5% de la population (n=27). Les patients ayant un revenu inférieur au Salaire Minimum Interprofessionnel Garanti (SMIG) représentaient 26% de la population et le revenu mensuel moyen était de 100.000 FCFA. La plupart des patients (84,7%) ne disposaient pas d´une couverture sanitaire. Un antécédent de diabète de type 2 était noté chez 15 patients (20,8%). La notion de tabagisme avait été rapportée chez 12 patients (16,7%) et 18 patients (25%) signalaient une consommation d´alcool. Un seul patient avait rapporté un antécédent familial de cancer du pancréas chez un parent du deuxième degré.

### Diagnostic clinique

**Figure 1:**
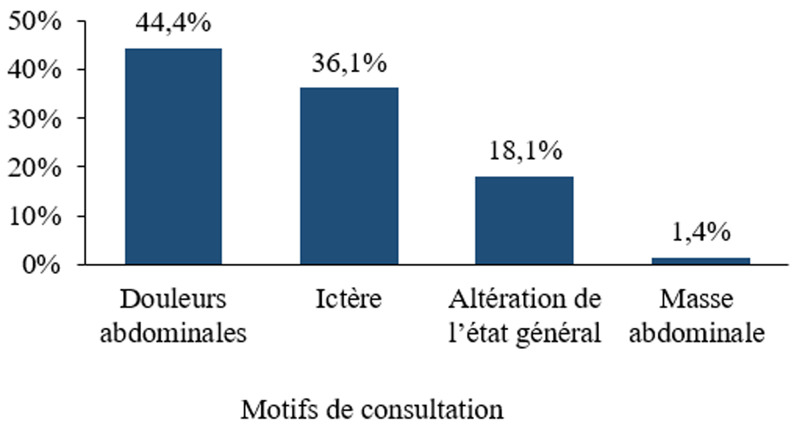
distribution des patients atteints de CP selon les différents motifs de consultation

Le délai moyen de consultation était de 3 mois. Les douleurs abdominales, l´ictère et l´altération de l´état général étaient les motifs les plus fréquents de consultation : respectivement dans 44,4%, 36,1%, et 18,1% des cas (Figure 1). L´amaigrissement était le signe clinique le plus fréquent (98,6%), suivi de l´ictère (79,2%) et de douleur abdominale (65,3%) siégeant le plus souvent en région épigastrique ou dans l´hypocondre droit (Tableau 1). L´examen physique à l´entrée notait une hépatomégalie chez plus de la moitié des patients (58,3%). La vésicule biliaire était palpable chez 26,1% des patients. Une masse épigastrique était notée chez 16 patients (22,2%) (Tableau 2). Par ailleurs, une altération de l´état général, à des degrés divers, était observée chez la grande majorité des patients (Indice de Performans Status selon l´Organisation Mondiale de la Santé IPS-OMS ≥ 2 dans 60 cas; 83,3%).

**Tableau 1:** répartition des patients atteints de cancer du pancréas selon les signes fonctionnels et généraux (n =72 cas)

	Effectifs (n)	Pourcentage (%)
Amaigrissement	71	98,6
Ictère	57	79,2
Urines foncées	55	76,4
Selles décolorées	52	72,2
Douleurs abdominales	47	65,3
Prurit	46	63,9
Vomissements	16	23,6

**Tableau 2:** répartition des patients atteints de cancer du pancréas selon les signes physiques (n=72 cas)

	Effectifs (n)	Pourcentage (%)
Hépatomégalie	42	58,3
Grosse vésicule	21	26,1
Masse épigastrique	16	22,2
Ascite	13	18,1
Adénopathies sus-claviculaires gauches	06	8,3
Circulations veineuses collatérales	5	6,9

### Diagnostic paraclinique

Biologiquement on notait un syndrome de cholestase dans 92,5% des cas, un syndrome de cytolyse dans 87,2%, et une hyperbilirubinémie totale dans 79% des cas (et dans tous ces cas la bilirubine conjuguée prédominait), une hyperglycémie dans 50,9%, une hypoprothrombinémie chez 65,4% des patients, corrigée après l´administration parentérale de vitamine K1 (Test de Koller positif) chez 91,2% des patients. Concernant les marqueurs tumoraux, leur dosage n´était pas systématique. On notait une élévation du taux du CA19-9 dans 68,4% des cas (13/19) et celui de l´ACE dans 62,5% des cas (10/16). Le diagnostic du cancer du pancréas a été posé sur la base des arguments échographiques et scannographiques chez 43 patients, scannographiques seuls chez 19 patients et échographiques seuls chez 9 patients (ces neuf patients n´avaient pas eu les moyens de faire un scanner thoraco-abdomino-pelvien). Le scanner abdominal a été réalisé chez 63 patients, et identifiait une tumeur pancréatique de taille variable dans la quasi-totalité des cas (n = 62; 98,4%), avec métastases hépatiques dans 25 cas, une dilatation du conduit pancréatique principal dans 37 cas et une dilatation des voies biliaires dans 48 cas. L´efficacité du diagnostic échographique était moins nette. Elle a été réalisée chez 52 patients et a permis d´objectiver une tumeur du pancréas chez 35 patients (67,3%) ainsi que des métastases hépatiques chez 06 patients. Après classification TNM, 51,4% des patients étaient au stade IV. La preuve histologique du cancer du pancréas a été obtenue chez 11 patients soit dans 15,1% des cas. L´adénocarcinome est la forme histologique retrouvée chez tous les patients.

### Aspects thérapeutiques

Un traitement symptomatique a été effectué chez la plupart des patients de notre étude et a consisté essentiellement en l´administration d´antalgiques pour la gestion de la douleur (88,9%; n = 64). Le traitement chirurgical était indiqué chez 27 patients soit un taux d´opérabilité de 37,5%. Cependant, 2 patients étaient éligibles pour une chirurgie curative soit un taux de résécabilité de 2,7%. Seul un patient a bénéficié d´un drainage biliaire par voie endoscopique par mise en place d´une prothèse biliaire plastique après une évacuation sanitaire en France. La chimiothérapie a été administrée chez 10 patients (13,9%). Dans 9 cas sur 10 elle était instaurée à visée palliative et dans un seul, il s´agissait d´une chimiothérapie adjuvante après une duodéno-pancréatectomie céphalique (DPC). Le protocole prescrit était la gemcitabine chez 7 patients et chez les 3 autres FOLFIRINOX (association 5-Fluoro-uracile, Acide Folinique, Irinotécan et Oxaliplatine). Le somme moyenne déboursée par les patients pour la prise en charge était de 955.882,4 FCFA soit 23,9 fois le SMIG, avec extrêmes de 200.000 et 3.000.000. FCFA.

### Aspects évolutifs et pronostiques

**Figure 2:**
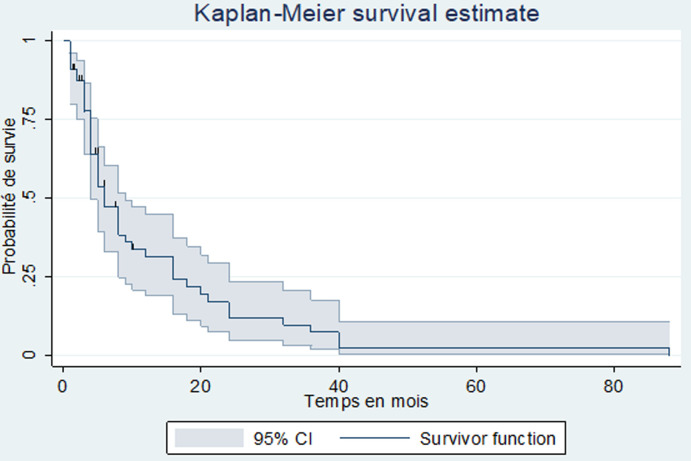
courbe de survie globale des patients atteints de CP

**Figure 3:**
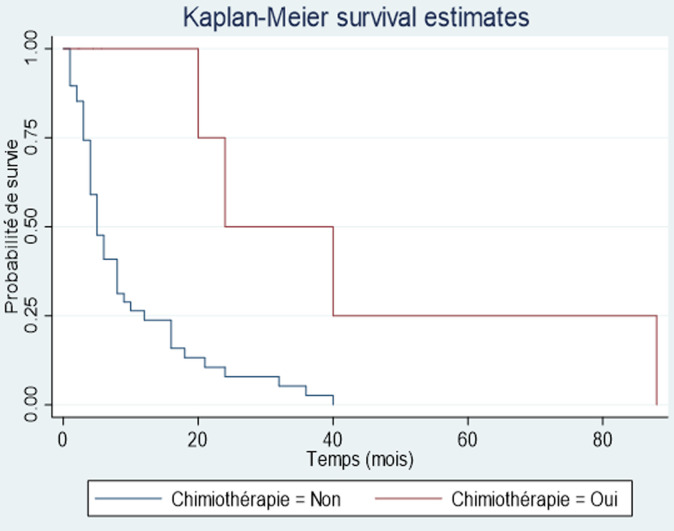
comparaison de la survie globale des patients traités par chimiothérapie versus patients non traités

**Figure 4:**
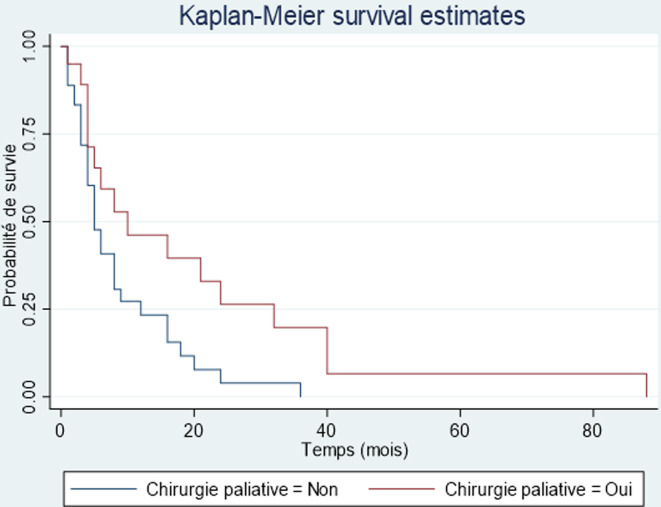
comparaison de la survie globale des patients opérés versus patients non opérés

Après le diagnostic, 11 patients étaient perdus de vue et donc exclus de l´étude de la survie. Le nombre de décès enregistré était de 53 parmi les 61 patients chez qui l´information était disponible, soit une létalité de 86,9%. La médiane de survie était de 6 mois avec un intervalle de 4 à 9 mois. Le taux de survie globale dans notre étude, selon la méthode de Kaplan-Meier était de 31,4% à un an, de 12,1% à deux ans, nulle à cinq ans (Figure 2). Parmi les facteurs influençant la survie des patients atteints de CP nous avons trouvé, en analyse univariée, la chimiothérapie (p = 0,023) et la chirurgie palliative (p = 0,0221). On notait par rapport à la survie globale un gain de 4,75 mois pour les patients ayant eu de la chimiothérapie (Figure 3) et de 1,25 mois pour les patients ayant eu une chirurgie palliative (Figure 4).

## Discussion

### Epidémiologie

La fréquence hospitalière du cancer du pancréas dans notre étude était 0,5%, avec 7,2 cas par an. Ce taux est probablement une sous-estimation de la fréquence réelle des cancers du pancréas au CNHU-HKM. En effet, certains malades n´avaient pas été inclus en l´absence d´éléments diagnostiques et de nombreux dossiers n´ont pas été retrouvés ou étaient incomplets donc inexploitables pour l´étude. L´âge moyen des patients de notre étude était de 59 ans. A contrario, les moyennes d´âges rapportées dans la plupart des études en Occident étaient beaucoup plus élevées. En effet, Maire *et al*. en France rapportaient une moyenne d´âge de 71 ans [6]. Notre étude confirme donc que de manière générale le CP est une pathologie du sujet âgé. Les variations observées selon les zones géographiques pourraient être dues à des facteurs génétiques encore méconnus propres aux populations noires. La prédominance masculine a été notée dans notre étude, avec une sex-ratio de 1,50. Plusieurs études ont rapporté, dans des proportions variables, cette prédominance masculine [7, 8]. Cela pourrait s´expliquer par le fait que le tabagisme, principal facteur de risque du cancer du pancréas, ainsi que la consommation d´alcool sont plus répandus chez les hommes que chez les femmes [9].

Les patients ayant un revenu inférieur au SMIG représentaient 26% de la population et le revenu mensuel moyen était de 100.000 FCFA. La plupart des patients (84,7%) ne disposaient pas d´une couverture sanitaire. Le coût moyen de la prise en charge était estimé à 955.882 FCFA soit 23,9 fois le SMIG. Ces données mettent en lumière la faiblesse du pouvoir d´achat d´une large frange de la population qui contraste avec le coût élevé de la prise en charge et pourraient expliquer le retard au recours aux soins modernes. Outre l´âge avancé et le sexe masculin, les autres facteurs de risque de cancer du pancréas répertoriés dans notre étude étaient l´antécédent de diabète de type 2, la consommation de tabac et d´alcool. L´antécédent de diabète de type 2 était noté chez 20,8% des patients. La consommation d´alcool et de tabac était effective dans respectivement 25% et 16,7%. Cette proportion de sujets tabagiques reste inférieure aux 21,4% et 35% rapportés respectivement par Hadizadeh *et al*. en Iran [10] et Zheng en Chine [11]. Cette différence pourrait s´expliquer par le taux de tabagisme plus élevé dans la population générale de ces pays industrialisés qu´au Bénin.

### Aspects diagnostiques

Le délai moyen de consultation était de 3 mois. Dans l´étude de Sidibé *et al*. [12] au Mali, ce retard était plus patent avec un délai de consultation moyen de 4,88 mois. Cependant, Jooste *et al*. [13] en France notaient un délai plus court d´un mois. La raison de cette différence est que nos patients s´orientent dans un premier temps vers des traitements traditionnels par défaut de moyens financiers ou du fait de leur perception de l´origine de la maladie (surnaturelle). La douleur abdominale était le principal motif de consultation retrouvé dans notre étude (44,4%), bien que la majorité de nos patients (84,5%) présentait une tumeur de la tête du pancréas qui se manifeste au début par un ictère sans douleur. L´apparition de la douleur est évocatrice d´une tumeur non résécable par envahissement cœliaque et associée à un mauvais pronostic. Ce résultat pourrait s´expliquer par le fait que les patients attendent l´apparition d´une symptomatologie douloureuse avant de consulter. Aussi, la survenue brutale de la douleur qui contraste avec l´installation insidieuse de l´ictère pourrait expliquer le recours aux soins plus spontané pour ce symptôme. L´échographie abdominale avait mis en évidence une masse pancréatique associée ou non à une dilatation des voies biliaires et du canal pancréatique dans 67,3% des cas. Ce résultat est nettement inférieur aux 90% rapportés par Coulibaly *et al*. [14] au Mali et aux 91,2% rapportés par Ashida *et al*. [15] au Japon. Cette disparité entre nos résultats et ceux rapportés dans ces deux travaux pourrait être due au fait que toutes les échographies n´étaient pas réalisées dans des centres de références par un personnel qualifié. Cela confirme par ailleurs le caractère opérateur-dépendant attribué à l´échographie. Le scanner abdominal avait permis d´identifier une masse pancréatique dans 98,4% confirmant ainsi la spécificité du scanner abdominal dans le diagnostic du cancer du pancréas.

Le diagnostic histologique a été obtenu chez 11 patients (15,1%). Hwang *et al*. [16] en Corée du Sud rapportaient un taux de confirmation histologique de 49,3%. Le type histologique le plus fréquent était l´adénocarcinome confirmant les données de la littérature. Le faible taux de confirmation histologique observé dans notre étude pourrait être dû à de l´indisponibilité de l´échoendoscopie et de spécialistes formés pour la réalisation de biopsies guidées en raison de la localisation profonde dans l´abdomen du pancréas le rendant peu accessible pour des biopsies transpariétales.

### Aspects thérapeutiques

La résection tumorale reste le standard de traitement pour le CP [4]. Cependant, seuls deux patients de notre série en avaient bénéficié soit un taux de résécabilité de 2,7%. Ce faible taux de résécabilité peut s´expliquer par le retard diagnostique plus prononcé dans notre contexte africain subsaharien qui limite les options thérapeutiques. Aussi, notons que la chirurgie pancréatique et hépatobiliaire est encore peu pratiquée dans nos hôpitaux car elle nécessite un plateau technique relevé (imagerie préopératoire, réanimation adaptée, instruments pour transsection parenchymateuse). Ce faible taux de résécabilité rapporté laisse une place non négligeable à la chirurgie palliative. En effet, 37,5% des patients de notre étude en avaient bénéficié contre 68,9 % rapportés par Imorou *et al*. [17] dans le service de Chirurgie Viscérale du CNHU-HKM. Cette différence pourrait s´expliquer par le fait que cette dernière étude a été réalisée uniquement un service de chirurgie. Notre étude avait inclus des patients pris en charge dans des services médicaux notamment en soins palliatifs qui pour la plupart n´étaient pas opérables.

Le traitement médical avait été administré chez la totalité des patients de notre étude. Il était surtout basé sur l´administration de vitamine K1 dans les hypoprothrombinémie et d´antalgiques pour la gestion de la douleur conformément aux recommandations de l´OMS. L´application de cette dernière mesure a été considérablement facilitée par l´installation de l´unité de soins palliatifs au CNHU-HKM de Cotonou qui dispose d´un équipement de production de sirop de morphine au niveau de la pharmacie de l´hôpital. La chimiothérapie était administrée chez 10 patients de la population soit 13,9%. En effet, l´état altéré au stade avancé du cancer n´autorise pas souvent l´administration de la chimiothérapie. Cependant, même dans les cas où la chimiothérapie est indiquée, la perception socio-culturelle de ce moyen thérapeutique et surtout les coûts élevés des anti-cancéreux entièrement à la charge des patients ne permettent pas une réelle mise en œuvre du traitement et une bonne observance.

### Aspects pronostiques et évolutifs

La mortalité dans notre série était très élevée (86,9%). La mortalité hospitalière était de 68,8%. La médiane de survie était de 6 mois avec un intervalle de 4-9 mois. La survie dans notre étude selon la méthode de Kaplan-Meier à un an était de 31,4%, à deux ans de 12,1% et à cinq ans, nulle. En France, Cowpply *et al*. [2] rapportaient une survie à 5 ans de 9%. La faible survie constatée dans notre série pourrait s´expliquer par le retard de recours aux soins qui implique un diagnostic à un stade de la maladie où les possibilités thérapeutiques ne sont que palliatives. Ces possibilités thérapeutiques sont d´autant plus limitées que les dépenses liées à la prise en charge des patients demeurent essentiellement à leur charge ou à celle de leurs familles. Enfin, il est important de signaler que la déficience du plateau technique de nos hôpitaux ne favorise pas une prise en charge optimale. Parmi les facteurs influençant la survie des patients atteints de CP nous avons trouvés en analyse univariée, la chimiothérapie (p = 0,023) et la chirurgie palliative (p = 0,0221). On notait par rapport à la survie globale un gain de 4,75 mois pour les patients ayant eu de la chimiothérapie et de 1,25 mois pour les patients ayant eu une chirurgie palliative. Kim *et al*. [18] au Canada avaient trouvé un lien statistiquement significatif (p < 0,0001) avec un gain de 5,6 mois par rapport à la survie globale. Ces chiffres confirment les données de la littérature qui stipulent que la chimiothérapie apporte un bénéfice de survie quels que soient les statuts T, N et R [19, 20].

### Limites de l´étude

La principale limite de ce travail est que le diagnostic histologique du cancer n´a été fait que dans 15% des cas de notre échantillon. Cela est lié au fait que dans la plupart des cas, la tumeur n´est pas résécable, et donc il n´y avait pas de pièce opératoire à analyser. Par ailleurs, nous ne disposons pas de possibilité de biopsies des lésions du pancréas sous contrôle échoendoscopique. Cette absence de prévue endoscopique chez la majorité des patients rend problématique le rôle de la chimiothérapie puisque celle-ci devrait être adaptée au type histologique. De fait, même si en analyse univariée la chimiothérapie palliative et la chirurgie palliative apparaissent associées à la survie, la non prise en compte du type histologique constitue un biais de confusion. Il convient donc dans notre pays d´améliorer le plateau technique et d´inciter des hépato-gastroentérologues à se former à l´endoscopie interventionnelle (échoendoscopie pancréatique, pose de prothèse biliaire) en vue d´une meilleure prise en charge des cancers du pancréas.

## Conclusion

Le cancer du pancréas est une pathologie d´évolution insidieuse dont le diagnostic est souvent posé à des stades tardifs. Il est redoutable, avec un pronostic péjoratif et une médiane de survie faible (6 mois). La chirurgie palliative et la chimiothérapie palliative améliorent la survie globale des patients. Cependant, le coût de la prise en charge élevé constitue un facteur limitant l´accès aux soins. Il s´avère nécessaire de sensibiliser la population et les professionnels de la santé sur cette affection en vue de la prévention et du diagnostic précoce. Il importe aussi de développer l´endoscopie interventionnelle en vue de moins recourir à la chirurgie palliative et disposer de preuve histologique par ponction-biopsie pancréatique sous échoendoscopie.

### Etat des connaissances sur le sujet

- Le cancer du pancréas est l´un des cancers les plus létaux dans le monde avec un ratio mortalité/incidence de 98%;
- Il représente la 5^e^ cause de décès par cancer dans le monde et reste le cancer digestif dont le pronostic est le plus défavorable, avec un taux de survie globale à 5 ans de 7 à 8%.

### Contribution de notre étude à la connaissance

- Le cancer du pancréas touche des patients âgés de 59 ans en moyenne, avec une légère prédominance masculine;
- Le principal motif de consultation était la douleur abdominale et plus de la moitié (51,4%) des patients avaient une maladie métastatique au moment du diagnostic;
- La médiane de survie globale était de 6 mois; la mortalité était de 86,9%; la survie à un an de 31,4%, et à 5 ans, nulle.
